# Treatment of Knee Osteochondral Fractures

**DOI:** 10.3390/healthcare10061061

**Published:** 2022-06-08

**Authors:** Mihai Alexandru Cordunianu, Iulian Antoniac, Marius Niculescu, Gheorghe Paltanea, Anca Daniela Raiciu, Horatiu Dura, Norin Forna, Ioana Dana Carstoc, Mihai Bogdan Cristea

**Affiliations:** 1 Faculty of Medicine, Titu Maiorescu University, 67A Gheorghe Petrascu, RO-031593 Bucharest, Romania; alexandru@cordunianu.ro (M.A.C.); mariusniculescu@yahoo.com (M.N.); 2 Faculty of Material Science and Engineering, University Politehnica of Bucharest, RO-060042 Bucharest, Romania; antoniac.iulian@gmail.com; 3 Academy of Romanian Scientists, RO-050094 Bucharest, Romania; 4 Faculty of Electrical Engineering, University Politehnica of Bucharest, RO-060042 Bucharest, Romania; 5 S.C. Hofigal S.A, RO-042124 Bucharest, Romania; daniela_raiciu@yahoo.com; 6 Faculty of Medicine, Lucian Blaga University of Sibiu, RO-550169 Sibiu, Romania; ioana.carstoc12@gmail.com; 7 Department of Orthopedics and Traumatology, Gr. T. Popa University of Medicine and Pharmacy, RO-700115 Iasi, Romania; norin.forna@gmail.com; 8 Department of Morphological Sciences, Carol Davila University of Medicine and Pharmacy, RO-020021 Bucharest, Romania; bogdan.cristea@umfcd.ro

**Keywords:** knee injury, osteochondral fracture, pediatric surgery, permanent implant approach

## Abstract

Osteochondral lesions (OCLs) that are frequently encountered in skeletally immature and adult patients are more common than once thought, and their incidence rate is rising. These lesions can appear in many synovial joints of the body, such as the shoulder, elbow, hip, and ankle, occurring most often in the knee. The term osteochondral lesion includes a vast spectrum of pathologies such as osteochondritis dissecans, osteochondral defects, osteochondral fractures, and osteonecrosis of the subchondral bone. When considering this, the term osteochondral fracture is preserved only for an osteochondral defect that combines disruption of the articular cartilage and subchondral bone. These fractures commonly occur after sports practice and are associated with acute lateral patellar dislocations. Many of these lesions are initially diagnosed by plain radiographs; however, a computed tomography (CT) scan or magnetic resonance imaging (MRI) can add significant value to the diagnosis and treatment. Treatment methods may vary depending on the location and size of the fracture, fragment instability, and skeletal maturity. The paper reports a 14-year-old boy case with an osteochondral fracture due to sports trauma. The medical approach involved an arthrotomy of the knee, drainage of the hematoma, two Kirschner wires (K-wires) for temporary fixation to restructure anatomic alignment, and a titanium Herbert screw fixing the fracture permanently. The patient had a favorable postoperative outcome with no residual pain, adequate knee stability, and a normal range of motion. The mobility of the knee was fully recovered.

## 1. Introduction

The importance of adequate treatment for patients with lesions of the articular cartilage and the covering bone must be emphasized. Regarding the pediatric population, this problem is more sensitive because they have a high level of activity and need a suitable functioning of the joints. Knee osteochondral fractures are the most common meet in orthopedic practice. They can occur in the lateral aspect of the femoral condyle, the inferior aspect of the patellar median ridge, and the inferior medial patellar facet. The fractured fragment can vary from small to large portions of the articular surface [1].

In the case of osteochondral fractures, sometimes, it can be challenging to establish a diagnosis. Even if a large osteochondral fragment is separated from the bone, it may have only a small, osseous portion, visible on plain radiographs. For a better diagnosis and management, the CT scan and the MRI techniques have a crucial role in identifying these types of lesions, especially in differentiating the acute chondral injuries, which do not contain subchondral bone or osteochondritis dissecans from osteochondral fractures [2,3].

## 2. General Presentation of the Knee Osteochondral Fractures

Osteochondral fractures can be misdiagnosed as pure soft tissue traumas, but they need much more complex treatment because the damage to the bone has to be addressed. These lesions determine a joint degeneration, which conducts to osteoarthritis because articular cartilage has low self-repair properties. Osteochondral fractures are usually localized in the shoulder (Hill Sachs lesions), distal humerus and radius, wrist, knee, hip, and ankle. In the case of weight-bearing joints, the progression to arthritis is faster, so it is essential to identify and treat these fractures early [4]. In this paper, we focus our attention on knee osteochondral fractures as described in Section 1.

A worldwide incidence of osteochondral fractures placed at the low extremity of the body is very difficult to be estimated due to the absence of medical images at the injury moment and clearly defined symptoms. Kenedy et al. [5] conducted a cadaveric study for osteochondral fracture of the knee. Their main conclusion was that these fractures appear in the medial condyle area due to knee extension associated with a lateral rotation or a knee flexion combined with a medial rotation. In some cases, tibial plateau lesions are present, proving that ligament constraints of the knee are damaged, and the external force is applied across the joint, stressing the cartilage. Gianotti et al. [6] reported that the incidence of knee ligament injuries is about 1%, and anterior cruciate ligament (ACL) injuries appear in 45% of cases [7]. Johson et al. [8] and Lahm et al. [9] considered that the incidence of osteochondral fractures with ACL injuries might be approximately 80%. Patellar dislocation associated with osteochondral fractures is present in 70% of patients [10]. Terry et al. [11] investigated isolated osteochondral fractures in the knee. They noticed that an incidence of 4% in acute knee trauma exhibited full-thickness lesions, which are linked to a hyperflexion injury. Sometimes minor subluxation in the knee joint zone can lead to joint surface deterioration due to the unconstrained nature of the anatomic place [4].

The knee joint represents the most common site of osteochondral fractures. The primary injury mechanisms involved in osteochondral fractures consist of a direct hit applied to the lateral or medial aspect of the knee and a knee flexion-rotation injury associated with internal rotation on a fixed foot. The fracture is due to the contact between the tibia and femur or patella and the lateral femoral condyle for the so-called acute patellar dislocation. Regarding the signs and symptoms of this injury, the patients present at the clinic with knee swelling, difficulty in weight-bearing activities, and complaints of severe pain. During the clinical examination, the pain is the most severe in the medial patellar aspect and lateral aspect of the lateral femoral condyle. Moreover, limited flexion and extension of the knee are reported. After this type of injury usually occurs, a large hemarthrosis [2,3].

In order to fix different osteochondral fractures, various treatments can be applied. The spectrum of procedures in this pathology is broad and ranges from microfracture-marrow stimulation with autologous chondrocyte implantation to internal fixation of the lesion. In the treatment of osteochondral lesions, some of the most important aspects are represented by the localization of the fracture, the size of the fractured fragment, stability of the lesion, and symptom severity [12]. Patient age and activity level are the most important factors when an individualized treatment method is chosen. The non-surgical treatment can be taken into consideration when we are dealing with small fragments of 5 mm or less detected on magnetic resonance imaging (MRI) or computed tomography (CT) scan, which give no symptoms or are very unlikely to cause them and are not situated on the bearing surface of the joint. This treatment includes rest, nonsteroidal anti-inflammatory drugs, intra-articular injections, cast or orthosis immobilization, and physical therapy [13].

Although surgical interventions have favorable results, they also have complications and failures, so a non-surgical technique is desirable to avoid or delay the surgery.

A viable treatment option consists of injective treatments straight in the affected articulation that can restore the joint homeostasis to delay or avoid surgical treatments by allowing the direct delivery of the therapeutic agents to the joint [14].

International societies such as Osteoarthritis Research Society International (OARSI) and the European Society for Clinical and Economic Aspects of Osteoporosis, Osteoarthritis, and Musculoskeletal Diseases (ESCEO) recommend a non-pharmacologic treatment in case of osteochondral lesions as the first line. They included intra-articular hyaluronic acid (HA) injections as recommended treatments for knee osteochondral lesions by providing favorable outcomes for pain relief, stimulation of endogenous synthesis of hyaluronic acid, and extracellular matrix components by synovial fibroblasts. Moreover, a minimum of 3 weeks of hyaluronic acid injection positively affected tissue repair [15].

Another intra-articular treatment consists of platelet-rich plasma (PRP) injections. PRP is extracted by centrifugation from autologous blood. This technique determines the growth of platelet concentration by 10-fold. It contains around 1500 proteins that can release macrophages and growth factors helpful in reducing inflammation, removing necrotic tissue, and articular cartilage repair. It was successfully used to treat osteochondral lesions of the knee joint [16].

Both techniques have proven to be safe and more efficient than nonsteroidal drugs and different types of knee immobilization. Some studies suggest that both HA and PRP injections should be used when treating osteochondral lesions for a better outcome.

Although they have proven very efficient in treating osteochondral lesions in general, when talking about osteochondral fractures, this type of treatment is limited and can be applied only in certain situations, as discussed above. Surgical intervention and internal fixation of the fragment should be considered in the case of a lesion bigger than 5 mm with the cortical bone attached to the chondral surface and involving a weight-bearing area [2,3]. The surgical intervention can be performed through a classical open approach—arthrotomy or arthroscopy.

Arthroscopic evaluation can highlight the lesion, allow the articular cartilage inspection, and simultaneously diagnose and treat the lesion. This approach has gained popularity by being regarded as the gold standard in assessing osteochondral lesions, which are detrimental to arthrotomy techniques [17].

The thorough exploration of the joint that the arthroscopy provides allows the surgeon to explore and observe any other associated lesions, such as meniscal or cruciate ligament lesions. Arthroscopic surgery is less invasive than open arthrotomy, allowing the patient a faster recovery and a low complication rate, representing a vital aspect, especially in a highly active population segment such as children.

Arthroscopy has gained significant ground in recent years for three main reasons, which include the use of improved technology (high-definition cameras and screens), which permit better surveillance of the joint; small-sized instruments and implants, which are perfect when treating children; and an increased incidence of sports injuries responsible for osteochondral fractures. Although it has many advantages, the arthroscopy approach is more technically demanding than the classical approach and requires constant training. Moreover, arthroscopy was introduced later in treating pediatric patients than in adults, and the new surgical techniques are usually practiced on adults before they are adapted to pediatric patients.

When refixation is not possible along with fragment removal, other techniques such as microfracture of the subchondral bone (MF) and autologous chondrocyte implantation (ACI) can also be used [18,19]. The microfracture of the subchondral bone is made in order to harvest bone marrow mesenchymal stem cells to reconstruct the cartilage defect. This first-line treatment is characterized by a minimally invasive nature, relatively low cost, and limited surgical morbidity. Different studies proved that MF has a beneficial effect on the subchondral bone, resulting in fibrocartilage tissue formation at the lesion site [20]. Cell-based procedures such as autologous chondrocyte implantation are restorative treatments that generate tissue with properties similar to hyaline cartilage and can restore the knee’s natural functionality. ACI is a process suitable for treating cartilage defects caused by direct injury where the damaged area is surrounded by normal healthy cartilage. It is a two-step technique; the first step involves harvesting a small articular cartilage piece via arthroscopy from the patient’s knee, which is later sent to a specialized laboratory. The chondrocytes are isolated and expanded in number. After 6–8 weeks, they can be reimplanted in the patient’s knee. The second step is performed to deliver the new cells to the damaged knee area, and the chondral defect is covered with a periosteal patch or collagen membrane. Disadvantages such as periosteal patch hypertrophy and extensive suturing or cell leakage are observed. In most cases, a favorable outcome is obtained because the cells attach to the damaged bone, and reproliferating of the articular cartilage cells begins. In the case of a lesion bigger than 5 mm, attention should be devoted to the surgical restoration with hyaline cartilage, which is performed through chondrocyte transplantation and matrix-assisted autologous chondrocyte implantation (MACI). In MACI, cultured chondrocytes are inserted and grown in a matrix scaffold and fixed in the chondral defect with the help of fibrin glue. MACI received the Food and Drug Administration approval in 2017 [20]. The matrix scaffold can be manufactured from Hyalograft C (Fidia Advanced Biopolymers, Abano Terme, Italy) [21], type I/III collagen membrane (Genzyme) [22], or type I collagen membrane (Arthro Kinetics Biotechnology GmbH, Krems an der Donau, Austria) [23]. Although the success of the marrow stimulation technique is directly linked to location and lesion size (less than 2 cm^2^) [24], cell-based repair procedures are effective for large and bipolar lesions [25,26].

In some cases, activated bone marrow aspirate concentrate combined with scaffold implantation at the cartilage defect site can be used. Whyte et al. [26] prepared a hyaluronic acid-based scaffold in which they seeded mesenchymal stem cells. This medical implant was delivered via dry arthroscopy. Arthroscopic fluid was drained from the working articular space, and a valveless cannula was positioned into the working portal. Based on this technique, the inspection of patellofemoral lesions may be improved through retraction of the joint capsule and synovium. The perimeter of the lesion is checked through dry arthroscopy, and the scaffold can be inserted through the working portal into the defect. The position of the implant is visualized through arthroscopy and fixed using fibrin glue. An open technique must be chosen if a complete exam of the lesion is not possible via dry arthroscopy. Oh et al. [27] applied dry arthroscopy with a simple retraction technique for knee cartilage regeneration using human umbilical cord and mesenchymal stem cells. They used special equipment such as a retraction plate and CO_2_ gas to substitute the saline solution. Sadlik et al. [28] implanted a collagen matrix used for autologous matrix-induced chondrogenesis repair based on dry arthroscopy with the help of an inserter rod and a dedicated guide. It can be noticed that a dry arthroscopy device was used for scaffold insertion, and its setup also facilitates the fibrin glue application onto the lesion and matrix.

The fracture fixation options may vary, including Steinmann pins, cannulated or metal screws, headless screws, and K-wires. Fixations’ methods such as bioabsorbable implants that do not require a secondary surgery have increased in popularity. In the case of permanent implants, implant removal is recommended after the fracture healing process ends [2,3]. Another important treatment option consists of headless compression screws made of non-bioabsorbable materials and can be inserted beneath the superficial level of the cartilage. These implants do not require removal, but they give the patient a significant disadvantage of remaining with foreign material in their body for the rest of their lives. This is an important aspect that has to be considered, especially when treating children.

## 3. Biocompatible Implants Used in Pediatric Osteochondral Fracture Treatment

**Figure 1:**
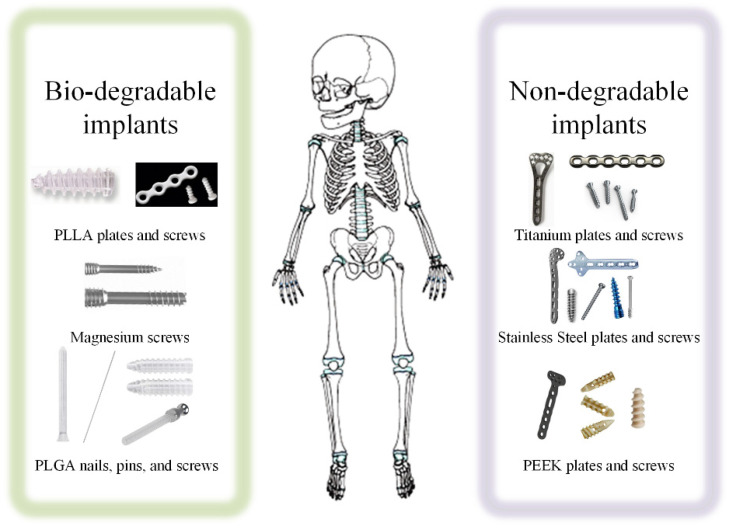
Biocompatible implants used in pediatric fracture treatment.

Biocompatible implants used in pediatric osteochondral fracture treatment could be permanent (made of stainless steel, titanium, or polyether ether ketone (PEEK)) or resorbable (poly-L-lactic acid (PLLA)-, poly-lactic-co-glycolic acid (PLGA)-, and Mg-based) (Figure 1).

**Figure 2:**
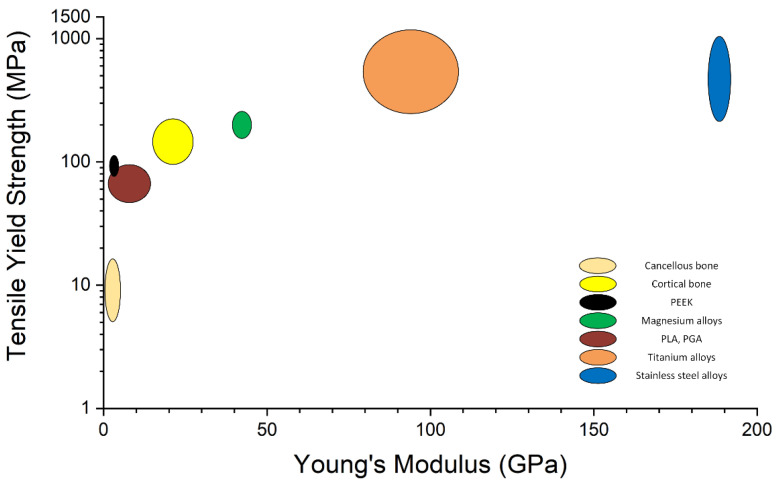
Ashby plot for different materials used in orthopedic implant manufacture.

The materials employed in the manufacture of surgical devices are important because their properties must be appropriate to the context in which they are used [29]. Regarding the surgical implants, one of the most important aspects consists of implant surface treatments made to improve its bioactivity and facilitate implant–tissue interaction, which leads to an accelerated bone healing process. The mechanical properties of the orthopedic implant material must have values appropriate to those of human bone (Figure 2). Despite many metallic implant options, in the case of pediatric patients are used only a few, from which increased attention is currently devoted to the bioabsorbable device class [30].

The most common metallic materials used in orthopedic surgery for permanent and temporary fixation are stainless steel and titanium, which require, in some cases, secondary removal surgery. The designed implants are used as temporary devices such as fracture plates, screws, wires, and hip nails or as permanent implants such as joint prostheses, with a supplementary recommendation of device removal when it is necessary [30]. Although stainless steel implants have been extensively employed in orthopedic surgery for decades, their popularity began to decrease because of their poor fatigue strength, which led to a rapid loosening of the implant. Moreover, MRI investigations that are not compatible with this type of material have contributed to the usage decrease [31]. Stainless steel wires were used to restore patellar fractures, and it was found that a risk of a secondary operation is present by comparing the situation with a group in which a non-absorbable polyester was involved [32]. A study with 50 participants investigated the metallosis on tissues and serum metal levels in children [33]. In the pediatric cohort, the main reasons to keep the implant in place are fracture after removal, high medical costs, increased morbidity due to surgical incisions, blood loss, and the inability to remove the implant entirely. An important study performed by Loder et al. [34] underlined that 41% of 372 respondents prefer to remove the asymptomatic stainless-steel implants from children almost every time, 36% say that they remove the implants only when it is necessary due to side effects, and 22% do not want to remove them. Pediatric orthopedists consider that the implant location is an important factor in deciding to keep or remove the implant. The group of non-pediatric doctors, which were included in this complex study, appreciates that implant removal should be performed in childhood after the healing of the fracture is ended. They found it difficult to treat patients who underwent orthopedic surgery and had stainless steel implants such as hip screws, epiphyseal fracture screws, and large implants for hip reconstruction or trauma. An important conclusion of this study consists of the fact that it is better to remove the implants when the patients are younger and likely healthy in order to hinder future complications.

Titanium-based alloys were initially used in aeronautics but gained ground in the medical field because of their corrosion resistance and high biocompatibility property. The naturally occurring oxide layer on the titanium surface improves the implant osseointegration process, making the alloy a very popular choice in the orthopedy field. Because bioinertia is an important drawback of the titanium alloys, to increase its bioactivity, surface treatments that facilitate the permanent implant osteointegration and decrease the risk of implant loosening are applied. Different approaches are taken to enhance the bone–implant interaction. One solution focuses on engineering the implant surface morphology and topography through machining, acid-etching, plasma spraying, grit-blasting, and anodization techniques, which offer increased surface energy and roughness, determining a better bone anchorage and osseointegration process. Another popular approach consists of bioactive surface development at the implant level through coating techniques. Through these methods, organic molecules such as enzymes, peptides, growth factors, or inorganic phases such as calcium phosphate are applied to the surface. Alloying titanium with different chemical elements is considered to improve bone–implant interaction, especially regarding the implant’s mechanical properties. Unfortunately, this method can alter the corrosion resistance of the metal due to the electrochemical reactions that emerge during the process [29,30]. New manufacturing techniques were developed to produce Ti implants to prevent bone cell overgrowth and provide antibacterial properties. Maher et al. [35] manufactured, using the 3D printing technique and electrochemical anodization, implants with vertically aligned titania nanotubes on the surface to reduce cell attachment and proliferation due to their hydrophobic nature.

Titanium Herbert screw used in fracture fixation is one of the most popular implants in pediatric orthopedy worldwide due to the increased biocompatibility of Ti and its relatively low cost regarding bioabsorbable implants made from polymers or biodegradable magnesium alloys. Currently, titanium alloys represent the material of choice in orthopedic implantology. The main material advantages are its good mechanical properties with values appropriate to human bone and the fact that it is lightweight. A significant advantage of titanium-based implants is that they are MRI compatible.

Polyether ether ketone (PEEK) permanent implants are used in pediatric orthopedy for foot surgery and anterior cruciate ligament reconstruction (ACL). The incidence of osteochondral fracture with ACL deterioration is very high in the case of children [4]. An extended analysis was performed by ACL study groups, which included a biennial survey regarding global trends in ACL reconstruction [36]. Regarding pediatric treatment, almost all the respondents (79%) considered delaying the surgical treatment of ACL injuries in children and adolescents very important. In the 10-year-old group, hamstring tendon (HT) autograft (75%) and quadriceps tendon (QT) autograft without bone (11%) were preferred. This cohort’s most used surgical techniques were transphyseal reconstruction, partial transphyseal technique, and all-epiphysial physeal-sparing fixation on the femur and tibia. In the femoral tunnel, PEEK interference screws are usually used [36]. PEEK is a polymer that provides artifact-free medical images when investigation techniques such as X-ray, CT, or MRI are used. New PEEK composite materials exhibit a bone-like modulus, and no stress shielding effects are present [37]. Repeated sterilization steps can be applied, and some PEEK composites [38,39] are characterized by high creep and fatigue resistance [40,41].

Bioabsorbable implants are increasingly used in orthopedic fracture management. Some important features of temporary orthopedic implants are proper mechanical properties, biocompatibility, adequate degradation rate, and dynamic corrosion. The medical visualization during the patient follow-up is also an important aspect of the applied treatment. The need for bioabsorbable implants has increased in the orthopedic field due to several factors.

Commercial bioabsorbable implants made from polymers were used for osteosynthesis [42,43]. Polymers such as poly-L-lactic acid (PLLA) or poly-lactic-co-glycolic acid (PLGA) are characterized by high brittleness, a low property of osteoconductivity, and a long degradation rate. A main drawback of the polymers-based implants is that they can be used only in non-load-bearing zones due to their low Young’s modulus and brittleness. Both materials, PLLA and PLGA, have a slow degradation rate, and inflammatory effects could appear. These types of material are used in osteochondral treatment fractures as sutures [44] or screws. Porcuitte et al. [45] conducted a prospective study made on 24 patients who suffered fractures treated through the osteosynthesis process based on small-diameter poly-L-lactide-poly-D-lactide (PDLLA) screws. Patients’ radiographs were regularly taken, and after a 1-year follow-up, no secondary displacement, instabilities, osteolysis, or growth disturbances were noticed. It was concluded that all the fractures were healed without complications.

Bioabsorbable metallic implants made from zinc (Zn), iron (Fe), or magnesium (Mg) alloys have been developed in the last few years [46,47]. Unfortunately, the low zinc mechanical strength and toxicity of the degradation products and the magnetic nature of iron and iron oxide degradation results led to additional research regarding Zn- and Fe-based alloys until a suitable solution can be developed. With their good biocompatibility, suitable Young’s modulus value, and increased ions rate release that promote bone healing and regeneration, the magnesium-based alloys emerged as the most promising biocompatible metal [48,49]. Magnesium is a natural component of the human body, exhibiting many advantages in orthopedic surgery such as lightweight, good compressive and tensile strengths, and Young’s modulus value closed to the one of the human bone (41 ÷ 45 GPa) [50,51]. Preclinical studies underlined that Mg-based alloys are beneficial to osteoblast development and plate chondrocytes growth [52,53]. From a mechanical properties point of view, the Mg screws exhibit higher pull-out forces than in the case of biocompatible polymers [53]. In the presence of body fluids, Mg alloys degrade through corrosion [47,54]. Secondary products such as oxides, hydroxides, or hydrogen species were noticed, but they were not associated with a clinical important side effect [55]. The Mg alloys’ corrosion produces a high amount of gas, and in some cases, rapid implant degradation is related to implant failure before fracture healing. It can be foreseen an increased need to control this process through surface treatments, and in this way, the osteogenesis can be enhanced [56,57]. On the other hand, a reduced degradation rate can result in fibrosis and significant inflammatory responses [58].

The main Mg-based alloys are classified into four types as follows: pure Mg, aluminum (Al) containing alloys that have secondary side effects such as neurological or liver disturbances, rare earth elements containing materials, and Al-free alloys [59,60]. Each alloying element is essential because some of them help to increase mechanical properties and corrosion resistance [61,62]. Higher biocompatibility and osteoconductivity can be achieved [63,64]. MgYREZr is the first approved material dedicated for clinical use in pediatric orthopedic surgery. From this alloy are manufactured pins, cortical bone screws, and Herbert screws [65]. These implants proved their efficiency in modified Chevron osteotomy, medial malleolar fracture [66], hallux valgus [67], and other types of fractures such as epiphyseal fractures, apophyseal avulsions, osteochondritis dissecans (OCD), tendon transposition based on interference screw use and displaced osteochondral fragments [68].

Jungesblut et al. [69] investigated 19 patients with a mean age of 13.7 years, of which 10 were females and 9 were males. The applied treatment consisted of an open or arthroscopic fixation of osteochondritis dissecans (OCD) lesions or displaced osteochondral fragments. A follow-up time of 6 months was set, and radiological investigations were regularly performed. The used implant was the MAGNEZIX^®^ Herbert type cannulate screw (MAGNEZIX^®^; Syntellix AG, Hannover, Germany). Complete healing was achieved for 12 patients, and one patient needed revision surgery due to intraarticular migration.

Mg screws are characterized by a stable fixation, no implant failure, no local pain or swelling, and there is no need for secondary surgery. Table 1 presents a retrospective analysis made by Baldini et al. [68] that includes children younger than 15 years old with different surgical procedures, treatments, and complications. In all the cases summarized there, resorbable MAGNEZIX^®^ screws (Syntellix AG, Hannover, Germany) were used.

Bioabsorbable implants can be safely used in load-bearing zones for about 12 months and do not require secondary surgery because of their biodegradability property.

An interesting case of a patellar dislocation was described in [68]. A 13-year-old boy suffered trauma when he was playing basketball. He was diagnosed with lateral dislocation of the left patella and a fracture. X-ray images were made before and after the patella reduction, and a preoperative CT scan was also performed. Due to the fact that the osteochondral fragment had a volume of 2.5 cm × 1.5 cm × 3.5 cm, an arthrotomic open reduction was chosen. Internal fixation was performed using three MAGNEZIX^®^ cannulated screws. A medial patella-femoral ligament reconstruction was performed. The fracture was reduced, and for its temporary fixation were used guide wires were and a 3.2 mm Herbert screw was introduced in a hole through low compression force. After 8 weeks post-surgery, the patient exhibited a complete range of motion and was full weight-bearing.

Gigante et al. [70] analyzed different intercondylar eminence fractures treated by resorbable magnesium screws. They presented a case series of patients with a mean age of 12.8. The surgical approach was arthroscopic reduction and internal fixation. Before the reduction, the arthroscopic evaluation of the menisci, articular cartilage, and ligaments was performed in all the selected cases. A K-wire was used to guide magnesium Herbert screws for temporary fragment reduction. The patients were evaluated at 1, 2, 6, and 12 months after the surgery. After 12 months of complete healing of the defect, screws’ resorption and their replacement with healthy bone were achieved in three cases.

## 4. Case Report

### 4.1. Clinical History

The reported case presents a 14-year-old boy that addressed the Orthopedic Emergency Department after a traumatic knee injury. The accident took place on the sports field while playing football, when suddenly, after a motion the patient cannot remember or precisely describe. However, it is supposed that he fell on the ground with his patella luxated. After the reduction in the luxation, the patient underwent knee radiographs.

**Figure 3:**
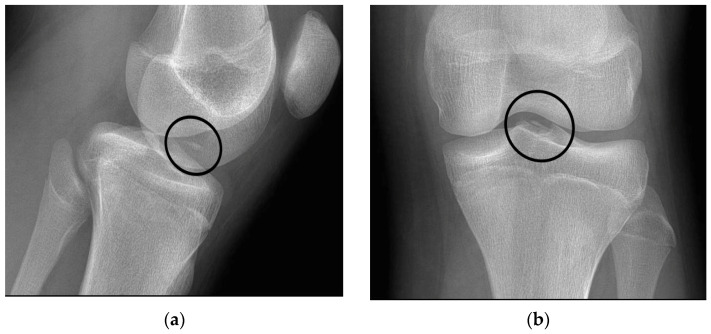
X-ray radiographs of the patient in two planes, (**a**) anteroposterior (AP) view and (**b**) lateral (L) view, of the injured knee. The fractured fragment is highlighted in the figure.

The radiographs showed the presence of an osteochondral fracture at the level of the injured knee (Figure 3).

**Figure 4:**
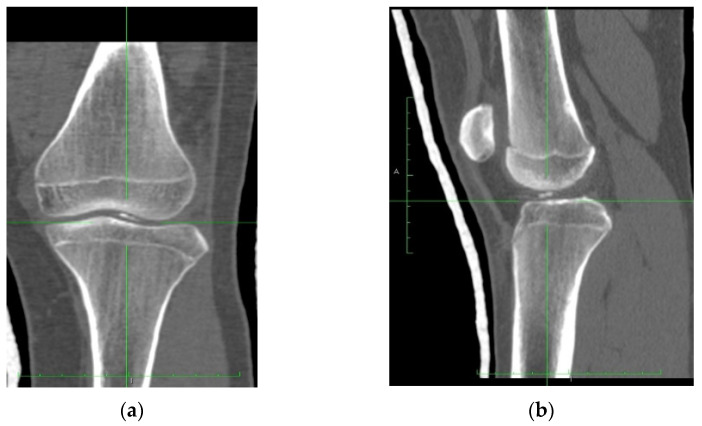
CT scans of the injured knee clearly showing the fractured fragment; (**a**) AP view and (**b**) L view.

The patient underwent a CT scan to understand better the fractured fragment’s position, size, and origin (Figure 4). Computed tomography high-resolution images were obtained, and the osteochondral fragment was precisely delimited and detected.

### 4.2. Surgical Technique

**Figure 5:**
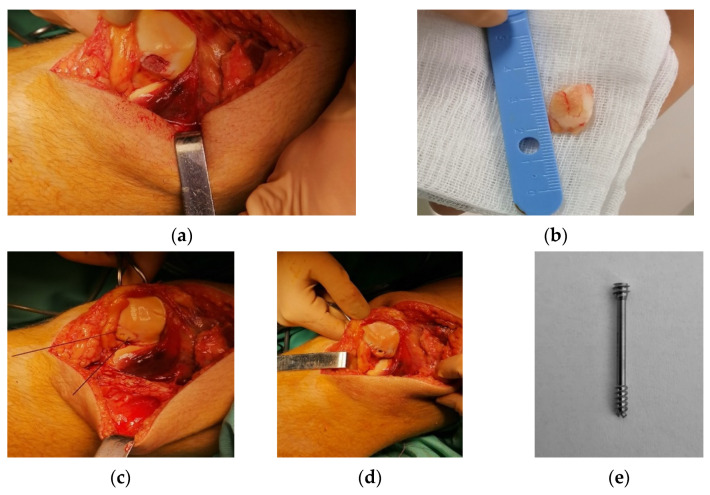
Details regarding the surgical techniques. (**a**) Highlighting of the patellar fracture; (**b**) the fractured fragment retrieved from the articulation and separated from the patient’s patella; (**c**) temporary fixation of the fractured fragment with K-wires; (**d**) internal fixation of the fractured fragment based on a headless titanium screw; (**e**) the titanium Herbert screw used for permanent fixation of the fracture.

The patient was put in a supine position (Figure 5a). The surgical approach to the knee articulation was made on the medial aspect of the patella. After the arthrotomy of the knee, the hematoma was drained. The fractured fragment of 1 cm from the patella was exposed (Figure 5b).

After the exposure, the fragment was temporarily fixed with two K-wires for an anatomic alignment with the rest of the patella (Figure 5c) until the permanent internal fixation was performed. For internal fixation of the fractured fragment, a headless non-bioabsorbable screw made from titanium alloy with dual threads that increase grip and create optimal compression was chosen. The head of the screw must be completely inserted into the bone to obtain optimal compression and avoid the possibility of soft tissue irritation or impingement (Figure 5d). Considering the dimensions of the fractured fragment, we opted for a Herbert screw of 24 mm length and 3.7 mm diameter to avoid comminution of the fractured fragment (Figure 5e).

An evaluation of the knee stability and range of motion was performed. The incision was closed without placing a drain, and the patient was treated with 3 days of postoperative administration of Ceftriaxone. The patient was discharged without any complications during the hospitalization.

After the surgical intervention, the patient was immobilized in a knee orthosis and walked with the help of crutches for eight weeks. In the first eight weeks, the goal was to protect the repair site and partially restore the normal quadriceps functions and patellar mobility. Moreover, in this period, the patient performed straight leg raises, quad sets, and ankle pumps. After the first eight weeks, gait training was emphasized initially, and weight-bearing status progressed as the patient gave up the crutches. Moreover, the patient received instructions for correct gait mechanics and reiterated as the normal gait pattern was restored. After the normal gait was restored, the patient was encouraged to swim and take walks in the park. Until week 12, the goals were to protect the surgical site and increase muscle endurance and joint stability.

After week 12, the patient started bike training while being careful with low-impact joint loading. The training with the bike lasted until week 16, after the surgery. After week 16, the patient could return to full normal activity, including playing sports.

During follow-ups, the knee’s function, inflammation, pain level, and stability were assessed by swipe tests, active and passive flexion and extension, passive knee hyperextension, anterior and posterior drawer test Lachman’s test, and the lateral and medial collateral ligaments assessment. The patient had no residual pain and recovered full mobility at the knee level.

**Figure 6:**
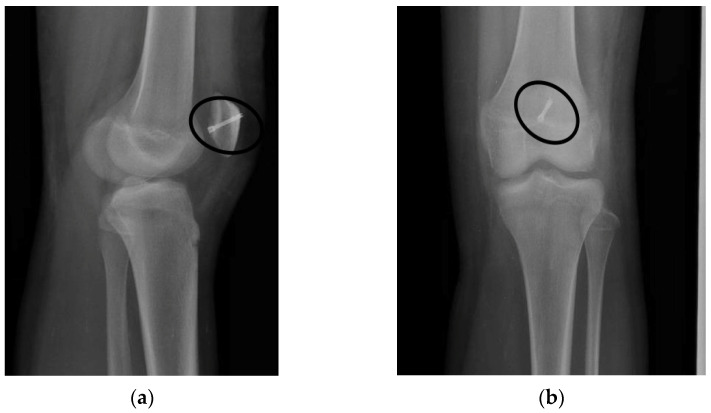
Follow-up X-ray radiographs of the patient in two planes, (**a**) L view and (**b**) AP view, of the injured knee. The medical images show that the fracture is healing and put in evidence a good tolerance of the screw by the surrounding tissues.

The X-ray follow-up medical images conducted one year after the surgical intervention (Figure 6) evidence the maintained reduction in the fractured fragment and showed no implant loosening effect. It can be concluded that the titanium implant exhibits a good osseointegration process in the patient’s bone, and it also stimulates new bone formation around it.

## 5. Discussion

Many injuries that occur in childhood are sports-related. The barriers and records that are valid in the case of adult trauma must be differently treated and investigated in the case of children and adolescents. We can currently notice an increased level of sports competitions, especially in the professional area, in which children tend to push too much their physical and mental boundaries. Although sports performances are on the rise, the anatomic structure of the human body remains the same, and the effects do not take long to appear. With modern imaging studies, osteochondral lesions in pediatric patients have been more commonly diagnosed in recent years. These lesions were pretty common decades ago, but there was a lack of imaging possibilities to analyze them.

Although they prove themselves as high-ranking athletes, we must not forget that we are dealing with children, and a child is not an adult in miniature because children’s skeleton has its particularities regarding the bone growth phenomenon. The particularities met, in this case, are responsible for the children’s injuries, which require typical treatments and dedicated implant use.

The clinical and surgical approach to the osteochondral lesion was presented in Section 2. Regarding the surgical treatment, three main steps must be underlined as follows: firstly, the fracture fragment has to be fixed. If the fixation is not an option, it is removed or based on regenerative techniques, in which bone marrow or chondrocyte stimulation is involved, and new bone formation occurs [71].

Titanium-based implants are adequate for young patients because titanium is a bioinert material, and over time it proves its efficiency. Moreover, it is compatible with MRI investigations. In the case of headless screws, such as the one used in our reported case, a secondary surgery for implant removal is not mandatory. However, some surgeons recommend it due to the difficulty of treating a problem in a region where there is already an implant. Unfortunately, when a permanent metallic implant is used, the risks associated with foreign body responses and biofilm formation must be considered, and antibiotic-based treatments should be applied when necessary to prevent important complications such as infections related to the implant.

For children, using such implants requires extensive care because they have an increased life expectancy and a high level of activity that predisposes them to repetitive trauma.

The current trends in osteochondral treatment fracture include bioabsorbable metal screws used for intra-articular fracture fixation and multiphasic scaffolds designed for osteochondral tissue engineering. The repair of the defects can be based on a tissue engineering approach, which mimics the physiological properties and the structure of the cartilage and bone. Osteochondral composites are used through press-fit into the defect without a supplementary fixation strategy. Multiphasic and gradient-based scaffolds were developed because the mono-phasic ones do not mimic the human body very well, and new tissue development is retarded [72]. Fabricant et al. [73] conducted a retrospective multicenter report in which they analyzed the fixation of traumatic chondral-only fragments in pediatric and adolescent athletes’ knees. They investigated 15 patients with a median age of 12.7 years. Six patients exhibited osteochondral fracture at the patella site, five patients had trochlea damage, and four young athletes presented lateral femoral condyle fracture. For all the patients, the injured sites were fixed with bioabsorbable implants (screws and tacks made from PLLA and absorbable suture manufactured from 6-0 braided polyglactin). Eight weeks after the surgery, one patient sustained a fall and needed a supplementary intervention for a dislocated fragment excision. A patellar stabilization surgery was necessary for another patient after 3.4 years postoperatively. The MRI images showed some excellent results, such as restoring the cartilage contour and reducing or absence of the subchondral edema. Unfortunately, one patient exhibited thinning of the cartilage, and another one had subchondral edema, fissuring, and cystic changes.

A future perspective for authors consists of biodegradable implant utilization in fracture fixation for the pediatric population. Currently, headless compression screws, made from bioresorbable materials such as polymers and magnesium, are considered viable options for treating different types of fractures. Mg-based implants exhibit a big advantage over polymer implants due to their higher stability [74] and osteoconductive properties [75]. One of the most used implants worldwide (see Table 1) in pediatric surgery is the Mg-based Herbert screw developed by MAGNEZIX^®^ CS of Syntellix AG. The alloy is based on the MgYREZr/WE43 system, containing 90% magnesium and a low percent of yttrium and zirconium. Cannulated screws (CS 2.7 and CS 3.2) permit a guidewire insertion. The screw models developed by MAGNEZIX^®^ have a self-cutting tip that makes their insertion very easy. The bone healing is usually not affected by the radiolucent zones, which are determined by the Mg screws. This phenomenon is based on the degradation and corrosion process of the Mg, and it disappears over time. Breakage of the MAGNEZIX^®^ CS during the bone healing process can be a direct consequence of the degradation [76].

In the case of different types of fractures, the Mg-based Herbert screws have proven their efficiency over titanium implants, but more studies need to be performed in this direction.

## 6. Conclusions

Titanium-based implants represent a reliable option for osteochondral lesion treatment in the pediatric population, and they offer certified good results. When considering the risks discussed above, it is important to search for new innovative solutions to develop bioabsorbable implants, which are considered patient-friendly and characterized by an increase in implant osteointegration.

The most used and discussed biodegradable material is magnesium. Magnesium implants are already approved by the Food and Drug Administration (FDA) to be used for humans, and some of the implants have Conformite Europeenne (CE) or Korean Drug Administration (KDA) certificates [49]. Biodegradable implants do not require implant removal surgery because a gradual degradation process occurs after a particular time.

Biodegradable implants are considered a safer option by comparing them with conventional devices because it is expected a faster recovery of the patient, which is an important aspect, especially in a highly active category such as children, and the risks associated with the permanent implant are avoided.

## Figures and Tables

**Table 1:** Children cases with different fractures and treatments based on resorbable MAGNEZIX^®^ screws [68].

Diagnosis	Age/Sex	Treatment and Follow up in Months	Complications
Tibial spine avulsion fracture	12/Female	Arthroscopic reduction internal fixation (ARIF) with two cannulated screws/26 months	None
Fracture–dislocation of patella	13/M	Open reduction internal fixation (ORIF) with three cannulated screws/18 months	None
Medial epicondyle avulsion	11/M	Open reduction internal fixation (ORIF) with two cannulated screws/14 months	Detachment of the screw head
Tibial distal epiphysis fracture	12/M	Closed reduction internal fixation (CRIF) (one all-epiphyseal cannulated screw and one trans-physeal Kirschner wire)/16 months	None
Flat foot and hallux valgus	8/F	Calcaneal notch filler (CNF) and hallux proximal physis emiepiphysiodesis (one cannulated screw)/12 months	None
Osteochondritis dissecans of the knee	14/M	Anterograde drilling and fixation (one cannulated screw)/12 months	None

## Data Availability

Not applicable.
